# Cancer-Associated Myeloid Regulatory Cells

**DOI:** 10.3389/fimmu.2016.00113

**Published:** 2016-03-29

**Authors:** Yannick De Vlaeminck, Anna González-Rascón, Cleo Goyvaerts, Karine Breckpot

**Affiliations:** ^1^Laboratory of Molecular and Cellular Therapy, Department of Biomedical Sciences, Vrije Universiteit Brussel, Brussels, Belgium; ^2^Centro de Investigación en Alimentación y Desarrollo, Hermosillo, Mexico

**Keywords:** cancer, tumor microenvironment, myeloid cells, monocyte, macrophage, dendritic cell, neutrophil, myeloid-derived suppressor cell

## Abstract

Myeloid cells are critically involved in the pathophysiology of cancers. In the tumor microenvironment (TME), they comprise tumor-associated macrophages (TAMs), neutrophils (TANs), dendritic cells, and myeloid-derived suppressor cells, which are further subdivided into a monocytic subset and a granulocytic subset. Some of these myeloid cells, in particular TAMs and TANs, are divided into type 1 or type 2 cells, according to the paradigm of T helper type 1 or type 2 cells. Type 1-activated cells are generally characterized as cells that aid tumor rejection, while all other myeloid cells are shown to favor tumor progression. Moreover, these cells are often at the basis of resistance to various therapies. Much research has been devoted to study the biology of myeloid cells. This endeavor has proven to be challenging, as the markers used to categorize myeloid cells in the TME are not restricted to particular subsets. Also from a functional and metabolic point of view, myeloid cells share many features. Finally, myeloid cells are endowed with a certain level of plasticity, which further complicates studying them outside their environment. In this article, we challenge the exclusive use of cell markers to unambiguously identify myeloid cell subsets in the TME. We further propose to divide myeloid cells into myeloid regulatory or stimulatory cells according to their pro- or antitumor function, because we contend that for therapeutic purposes it is not targeting the cell subsets but rather targeting their protumor traits; hence, myeloid regulatory cells will push antitumor immunotherapy to the next level.

## Introduction

The immune system’s role in malignancies appears to be more complex than originally anticipated (1–5). Several immune mechanisms can support antitumor immunity; however, they are often counteracted due to immunosuppressive mechanisms exerted by the tumor and its environment (6). For many cancers, including ovarian, renal cell, colorectal, and breast cancer, prognosis, metastatic burden, and therapeutic response rates have been linked to immune cell populations that infiltrate the tumor (7–10). Consequently, the focus in antitumor immunotherapy started to shift from exclusive stimulation of innate and adaptive immunity to combinations with intratumoral modifications (11).

The tumor microenvironment (TME) comprises a complex milieu of non-malignant cells, such as cells from endothelial, mesenchymal, and immunological origin (5). Among the tumor-infiltrating immune cells, myeloid cells represent a prominent component both in terms of quantity and function (12–14). They consist of a heterogeneous mixture of monocytes, tumor-associated granulocytes (mainly neutrophils or TANs), myeloid-derived suppressor cells (MDSCs), tumor-associated macrophages (TAMs), and tumor-associated dendritic cells (TADCs). To complicate matters, these different cell types are characterized by different polarization states with both stimulatory and tolerogenic functions, often referred to as type 1 and type 2 states, respectively. It is well described that mature TADCs, type 1 TAMs and TANs can counteract tumor growth by stimulating T-cell-mediated antitumor immunity (15). By contrast, type 2 TAMs and TANs, immature TADCs, a subset of mast cells and MDSCs mainly promote tumor progression via immunosuppression, stimulation of angiogenesis, and secretion of growth factors. Consequently tumor-infiltrating myeloid cells are critical contributors to the “never-healing-wound” that characterizes most solid tumors and as such they are an attractive target for novel therapies (16–20). However, interpreting relevant literature is currently challenging since the same cell type is often given another name based on the use of the so-called “unique” markers and functions. In reality, these markers and functions are shared between different cell types as they are driven by tumor-derived factors that trigger transcriptional programs and as such determine the cell’s phenotype and activity.

## Identity Crisis

Under physiological conditions, myeloid cells can be distinguished by their ontogenic transcription factors. Monocytes, macrophages, and DCs are derived from the monocyte/macrophage and DC precursor, while granulocytes originate from the granulocyte precursor. Both precursors arise from the granulocyte/macrophage progenitor, which in turn is derived from the common myeloid progenitor stemming from the hematopoietic stem cell (21–23).

**Figure 1:**
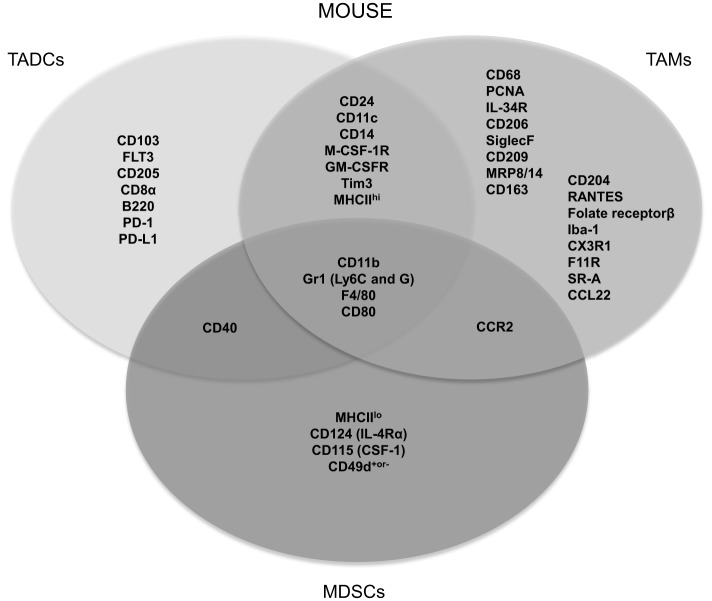
**Most frequently used specific and shared surface markers for the differentiation of murine TADC, TAM and MDSC**.

Myeloid cells arising in cancer patients fail to display normal features of myeloid differentiation resulting in atypical myelopoiesis in the bone marrow, periphery, and within the TME (10). In general, modulatory signals originating from the TME, such as transforming growth factor (TGF)-β, colony-stimulating factor-1 (CSF-1), CSF-2, and various others, induce the attraction and expansion of bone marrow and blood-derived immature myeloid cells. Once in the tumor, they are classified as monocytes, TAMs, MDSCs, TADCs, and TANs, based on their surface marker expression, which can differ depending on the tumor type, the location within the tumor as well as the stage of tumor progression (24). Consequently, markers that are often used to distinguish murine myeloid cell subsets, such as CD11c, F4/80, CD11b, and Gr1, appear to be shared among subsets as illustrated in Figure 1.

Perhaps one of the most striking examples of shared markers that are often used to define myeloid cell subsets is the expression of the prototypical DC and macrophage markers CD11c and F4/80, which are both expressed by TADCs and TAMs (15, 25, 26). The same holds true for human tumor-infiltrating myeloid cells, as, for example, human TAMs are often characterized by CD68, a receptor that is also expressed by other stromal tumor populations (27). It is clear that myeloid cells within the TME are not only hard to distinguish from each other but they can moreover trans-differentiate into one another. In the case of MDSCs, for example, it has been shown that they could trans-differentiate into macrophages, granulocytes, or DCs (28–35). Furthermore, it was also suggested that TADCs can converse into morphological, phenotypical, and functional TAMs (36). However, we must be careful to draw decisive conclusions from this study as they made use of CD11c, CD11b, and F4/80 as discriminating phenotypic markers. As mentioned above, these are expressed on both TAMs and TADCs, albeit at different levels.

The above examples suggest that it seems more appropriate to state that the formerly applied terms to characterize tumor-infiltrating myeloid cell subpopulations represent extremes of a continuum in a universe of activation states, including classically and alternatively activated immature, mature, type 1 and type 2 tumor-infiltrating myeloid cells (37, 38).

## The Type 1/Type 2 Paradigm

Although myeloid cells can be subdivided based on specific ontogenic, functional, and phenotypic features, they are all subjected to the same “ground rules” present within the TME in a stratified way (39). This results in the adoption of two main polarization states that mirror the T helper 1/2 paradigm of CD4^+^ T cells.

Myeloid cells with a type 1 phenotype function as inflammatory, tissue (matrix)-destructive and immune-stimulating cells. These cells perform their immune stimulatory function by secretion of pro-inflammatory cytokines, such as tumor necrosis factor (TNF)-α, interleukin-1 (IL-1), IL-6, and IL-12 combined with a strong capacity to process and present tumor antigens. Furthermore, they are able to kill tumor cells by the production of inducible nitric oxide synthase (iNOS) (40). These functions are metabolically supported by increased glycolysis and pentose phosphate pathway, which provide rapid ATP production and biosynthetic intermediates required for the generation of pro-inflammatory proteins (41, 42). Alternatively activated type 2 tumor-infiltrating myeloid cells are characterized as cells mediating tissue repair and immune suppression. To exert their suppressive function, type 2 tumor-infiltrating myeloid cells secrete among others IL-10 and TGF-β, and express enzymes, such as arginase-1 and indoleamine 2,3-dioxygenase (IDO), which deplete the TME from nutrients essential for T cells. Moreover, type 2 tumor-infiltrating myeloid cells produce factors involved in tissue repair and angiogenesis, such as vascular endothelial growth factor (VEGF) (43). Finally, they tend to rely on oxidative phosphorylation, which is important for their long-term activation and subsequent return to homeostasis (41).

In the case of TANs and TAMs, this is reflected in the N1/N2 and M1/M2 paradigm. Of note, it is described that TAMs have a transcriptional profile distinct from the classically activated M1 or alternatively activated M2 macrophages found under tumor-free steady-state conditions. However, due to a certain “overlap” of transcriptional molecules present in the classically and alternatively activated macrophages and the TAMs, it has been suggested that TAMs can adapt an M1, M2, or shared M1/M2 signature (44). For example, human TAMs co-expressing HLA-DR, a typical M1 marker as well as CD163, a typical M2 marker have been observed as well; again reflecting that also the type 1/type 2 paradigm reflects two extremes of a continuum (45, 46). Likewise, TADCs and MDSCs seem to preserve their original division. Tumor-associated DCs, similar to peripheral DCs, are subdivided into plasmacytoid DCs (pDC), two conventional DC subsets (cDC1 and cDC2), and monocyte-derived DCs (47). MDSCs are subdivided into a monocytic subset and a granulocytic subset. While MDSCs are intrinsically characterized by suppressive type 2 functions, TADCs can display Janus-like features. When activated, they can initiate potent tumor antigen-specific immune responses. However, when immature, they contribute to genomic damage, angiogenesis, stimulation of tumor cell growth, and spreading. Moreover, immature TADCs induce antigen-specific T-cell unresponsiveness through direct and indirect mechanisms (48). The knowledge that TADCs present tumor antigen-derived peptides, however, fail to provide co-stimulation has instigated attempts to activate TADCs for immunotherapeutic purposes (49). Indeed, targeted delivery of DC potentiating stimuli, as exemplified by the delivery of TriMix mRNA, enables TADCs to migrate to draining lymph nodes and activate tumor antigen-specific T cells, which in turn migrate to the tumor to mediate tumor cell rejection (50).

It has been suggested to redefine MDSCs as “myeloid regulatory cells” based on their ability to suppress host antitumor immunity (51). However, since their functional, metabolic, and phenotypic features are not unique to MDSCs but shared between the suppressive “type 2” myeloid cells, we question the confinement of the term myeloid regulatory cells to MDSCs (52). In accordance with the type 1/type 2 paradigm, we propose to redevise tumor-infiltrating myeloid cells into tumoricidal myeloid stimulatory and tumor-promoting myeloid regulatory cells. Myeloid stimulatory cells would include polarization states with an M1, mature TADC, and N1 phenotype, while myeloid regulatory cells would include their type 2 counterparts: M2, immature TADCs, and N2, respectively, together with the monocytic and granulocytic MDSCs. Different tumor-infiltrating myeloid cell subsets amplify tolerance by cross-talk with each other and other stromal cells from the TME. For example, MDSCs induce type 2 TAMs via IL-10, which in turn enhance the IL-10 production by MDSCs, resulting in a positive feedback mechanism for sustained type 2 TAM generation (53). The myeloid regulatory cells and myeloid stimulatory cells represent a classification system based on shared functional hallmarks that result from common intratumoral incentives.

The functional skewing of myeloid cell populations in antitumor or myeloid stimulatory cells, and protumor or myeloid regulatory cells is possible due to myeloid cell plasticity. Myeloid cells adapt to environmental signals, which govern their transcriptional profile and consequently their phenotypical and functional traits. It is becoming clear that transcriptional pathways promoting polarized functions of different myeloid cell subsets share common constituents. Key signaling networks cooperate, integrate, and finally converge into a few pathways, such as that of the signal transducer and activator of transcription (STAT) family and nuclear factor-κB (NF-κB) to promote the protumor traits of different myeloid cell populations (54). Therefore, the plasticity of myeloid cells also challenges their categorization in subtypes based on phenotypic features. The observation that transcription factors and signature genes dictate their polarization and, therefore, function favors classifications based on stimulatory versus regulatory properties (26, 55). We believe that a simplified functional subdivision into myeloid stimulatory or regulatory cells gives a more perspicuous view on tumor-infiltrating myeloid cells. The scenario of shared phenotypical and functional traits driven by common transcriptional programs suggests that it is feasible to develop strategies to target different myeloid cell populations simultaneously and consequently therapeutically affect the protumor network established by cancer-associated myeloid cells.

## Implications for Cancer Research and Therapy

One of the joint hallmarks of myeloid cells is their plasticity. This not only makes them susceptible to tumor-derived cues but also enables their manipulation and repolarization. Subsequently numerous studies have been conducted to either block their recruitment, deplete one or more suppressive myeloid cell subsets, and/or repolarize type 2 subsets to more tumoricidal type 1 myeloid cells (Table 1). From the studies listed in Table 1, we can draw some general conclusions.

**Table 1:** **Overview of strategies studied to manipulate murine and human tumor-infiltrating myeloid cells**.

Target	Moiety	Cancer model	Effect	Reference
**Tumor-infiltrating myeloid cell-related receptors**				
CSF-1R/CSF-1 signaling	Small molecules, monoclonal Ab, siRNA	Mouse (melanoma, glioblastoma, breast, pancreas, and colorectal cancer)	↓TAM, Mo-MDSC recruitment	(25, 56–61)
			TAM, M-DCs depletion	
			↑TAM repolarization	
		Human (neuro- and glioblastoma, giant cell tumor, and lung cancer)	↓TAM recruitment	(62–66)
			↓CD14^dim^/CD16^+^ monocytes in plasma	
			TAM depletion	
CCL2/MCP-1	Small molecules and monoclonal Ab	Human (melanoma and prostate cancer)	↓TAM recruitment	(67)
CD11b	Monoclonal Ab	Mouse (ovarian cancer)	TAM, TADC, and MDSC depletion	(68)
IL12R/IL18R	Adenovirus	Mouse (sarcoma)	↑TADC repolarization	(69)
CD40	Monoclonal Ab	Mouse (bladder cancer)	↑DC activation	(70)
		Human (several cancers)	↑DC maturation	(71)
Retinoic acid receptor	Vit. A derivate	Mouse (cervical cancer)	↑Maturation of iMC	(72)
		Human (lung, renal cancer)	MDSC depletion	(73, 74)
TLR3,5 or 9	Agonists, siRNA, and TLR ligands	Mouse (breast, ovarian, and renal cancer)	↓MDSC	(75–78)
			↑TADC repolarization/maturation	
Gr1	Monoclonal Ab	Mouse (fibrosarcoma)	MDSC depletion	(79)
G-CSF	Monoclonal Ab	Mouse (several cancers)	↓Tumor-associated circulating myeloid cells	(80)
GM-CSF	Monoclonal Ab	Mouse (pancreas cancer)	↓Recruitment of Gr1^+^/CD11b^+^	(81)
IL6-R	Monoclonal Ab	Mouse (skin squamous cell cancer)	↓MDSC	(82)
cKIT/SCF	Monoclonal Ab	Mouse (colon carcinoma)	↓MDSC recruitment	(83)
Bv8	Monoclonal Ab	Mouse/human (several cancers)	↓MDSC recruitment and Expansion	(84)
Carboxy-N-glycan	Monoclonal Ab	Mouse (blood, breast cancer)	↓MDSC	(85)
DR3	Cytokine	Mouse (immature DC)	↑DC maturation	(86)
**Intracellular tumor-infiltrating myeloid cell regulators**				
Several miRNAs	Nanoparticles	Mouse (ovarian, breast, and lung cancer)	↑TADC, TAM repolarization	(87–90)
STAT3	Small molecules and siRNA	Mouse (melanoma and breast cancer)	↑TADC, TAM/MDSC repolarization	(91, 92)
		Human (peripheral blood and several tumors)	↓im. suppr. of MDSC	(93, 94)
Small Rho GTPases	Cytostatic drug	Mouse (lung cancer)	↓TADC formation	(95)
Tyrosine Kinase	Small molecule	Human (renal cell carcinoma)	↓MDSC	(96–99)
Legumain	Cytostatic prodrug	Mouse (breast, lung cancer)	TAM depletion	(100)
		Human (breast cancer)	↓im. suppr. of MDSC	
PDE5	Small molecule	Mouse (colon and breast cancer)	↓im. suppr. of MDSC	(101)
IRF8	Overexpression	Mouse (breast cancer)	↓MDSC accumulation	(102)
p50 NF-κB	siRNA	Mouse (melanoma, and pancreas cancer)	TAM repolarization	(103)
**Extracellular suppressive molecules**				
Lactate dehydrogenase	Small molecule	Human (melanoma, and prostate cancer)	↑TADC repolarization/maturation	(104)
VEGF	Monoclonal Ab	Human (colon, lung, and breast cancer)	↓Immature DCs	(105)
COX2	Small molecule	Mouse (breast cancer)	TAM repolarization	(106, 107)
		Human (blood samples)		
Fatty acid oxidation	Small molecule	Mouse (lung cancer)	↓ im. suppr. of MDSC	(94)
Phosphatidylserine	Monoclonal Ab	Human (prostate cancer)	TAM, MDSC depletion	(108)
			↑TAM, DC maturation	
ROS	Small molecule	Mouse (colon and lung cancer)	↓im. suppr. of MDSC	(109)
**Undefined targets**				
	Cytostatic drugs	Mouse (bone marrow-derived MDSC, lung, breast, and ovarian cancer)	↑MDSC diff. into DC	(110–112)
			↓MDSC	
	Peptibodies	Mouse (thymoma)	MDSC depletion	(113)
	*T. Gondii*	Mouse (ovarian cancer)	↑immune stimulatory DC	(114)
	Histidine-rich glycoprotein	Mouse (fibrosarcoma, breast, and pancreas cancer)	TAM repolarization	(115)

*CSF, colony-stimulating factor; Ab, antibody; siRNA, small interfering RNA; CCL2/MCP-1, chemokine (C-C motif) ligand 2, monocyte chemotactic protein 1; Vit A, Vitamin A; TLR, toll-like receptor; G-CSF, granulocyte colony-stimulating factor; GM-CSF, granulocyte-macrophage colony-stimulating factor; cKIT/SCF, stem cell factor; DR3, death domain-containing receptor-3; STAT3, signal transducer and activator of transcription 3; GTP, guanosine triphosphate; PDE5, phosphodiesterase type 5; IRF8, interferon regulatory factor 8; NF-κB, nuclear factor κB; VEGF, vascular endothelial growth factor; COX2, cyclo-oxygenase 2; ROS, reactive oxygen species*.

First, therapeutics are administered systemically in most studies. However, for most if not all targets, this can result in major immunological deficits in the long run as the myeloid cell compartment is of paramount importance for maintenance of immunological homeostasis outside the tumor tissue. Therefore, we propose to focus on the development of therapeutic delivery systems that can restrict depletion and/or modulation of myeloid cells to the tumor niche.

Second, it is remarkable to see how most studies tend to focus on the modulation of “one particular subset” while most often “shared” receptors and regulators are targeted, such as CD11b and CSF-1 (68). Therefore, we believe that it would be more interesting to evaluate the behavior of the general intratumoral myeloid regulatory cells’ state after therapy. For example, several groups that focus on TAMs have targeted CSF-1 and demonstrated a decrease in TAM recruitment, depletion, and/or M2 to M1 repolarization. Nevertheless, it would be more alluring to evaluate what CSF-1 blockage does with the shape of the general intratumoral myeloid regulatory cell population since CSF-1 is seen as one of the major attractants of “all” immature myeloid cells to the TME.

Third, instead of targeting “shared” phenotypic markers to block the recruitment or deplete a presumed tumor-infiltrating myeloid cell subset, we believe that targeting shared functional mechanisms can result in a higher chance that the whole TME can repolarize toward a myeloid stimulatory cell comprising milieu. Blockade of myeloid suppressive mechanisms can, therefore, be seen as a more “general” way to “target” the type 2 cancer-associate myeloid cell population without focusing on one particular subset. To that end, several strategies can be envisaged, such as blocking the VEGF and/or TGF-β pathways (19, 96). Blocking the TGF-β pathway is, to our current knowledge, one of the most appealing strategies to prevent polarization of type 2 tumor-infiltrating myeloid cells. This may have profound impact on the balance of M1–M2 TAMs and N1–N2 neutrophils and allow DC maturation. Blocking may also regulate the excessive recruitment of tumor-infiltrating myeloid cells and as such also decrease the amount of myeloid regulatory cells (19). Based on the lack of a unique definition for the tumor-infiltrating myeloid cell subset of interest, together with the observation that tumor-infiltrating myeloid cells play a multifaceted role and can exhibit tumoricidal capacities, it is reasonable to propose that re-polarization rather than their depletion will be substantially more beneficial (116). This is exemplified by a study where TGF-β blockade resulted in a therapeutic antitumor response caused by an influx of repolarized oncolytic TANs that expressed high levels of pro-inflammatory cytokines, while neutrophil depletion significantly blunted these antitumor effects (29). Also CSF-1R inhibitors, TLR9 ligands combined with anti-IL10R antibodies or histidine-rich glycoproteins resulted in an antitumor immune response caused by repolarization of the intratumoral myeloid regulatory cell population (56, 75, 115).

Fourth, also non-tumoral, non-immunologic stromal cell populations, such as endothelial cells and fibroblasts, can have a major impact on the composition of the tumor-infiltrating myeloid cells, implicating that also these cell types are important to consider when envisaging modulation of the tumor-infiltrating myeloid cell composition (5). For example, targeting endothelial cells using a VEGF inhibitor resulted in decreased numbers of intratumoral MDSCs, while the depletion of cancer-associated fibroblasts, which express many immunosuppressive molecules, showed impaired tumor growth (96, 117).

Finally, although murine *in vivo* and human *in vitro* studies show great promise for tumor-infiltrating myeloid cell combating therapeutics, there is a current lack of clinical data regarding the effects of such treatments on the tumor-infiltrating myeloid cells and more specifically the regulatory myeloid cell versus stimulatory myeloid cell ratio in cancer patients (118).

Important to keep in mind is that everything in biology depends on homeostasis. Repolarizing myeloid regulatory cells to type 1 tumor-infiltrating myeloid cells will also lead to imbalance, probably increased antitumor immunity but most likely also an enhanced state of chronic inflammation that in turn can induce the recruitment of MDSCs again (118). However, it is presumed that also antitumor immunity is allowed to take course and as such elimination of tumor cells is promoted. Therefore, one can postulate that the inflammation will resolve as soon as all tumor cells are rejected and tissue repair is complete.

## Concluding Remarks

In this perspective paper, we suggest that different myeloid cell populations evolve along with tumor progression, and that their phenotype and function is not as distinct as previously anticipated. This is supported by the plethora of papers on the subject of TAMs, TADCs, TANs, MDSCs, and how the plasticity of these cells allows them to acquire different activation states, even trans-differentiate into another cell subset, depending on the encountered factors. In the TME, a number of suppressive molecules trigger transcriptional programs that govern phenotypical and functional changes, endowing myeloid cells within the TME with a type 2 or immunosuppressive phenotype irrespective of the myeloid cell subset. We argue that the commonalities in phenotype and function provide an opportunity for therapeutic interventions that may concomitantly skew the myeloid cells to a type 1 state or as proposed to myeloid stimulatory cells.

## Author Contributions

YDV, AGR, CG, and KB contributed equally to the manuscript.

## Conflict of Interest Statement

The authors declare that the research was conducted in the absence of any commercial or financial relationships that could be construed as a potential conflict of interest.
